# Crop-ecology and nutritional variability influence growth and secondary metabolites of *Stevia rebaudiana* Bertoni

**DOI:** 10.1186/s12870-015-0457-x

**Published:** 2015-02-27

**Authors:** Probir Kumar Pal, Rajender Kumar, Vipan Guleria, Mitali Mahajan, Ramdeen Prasad, Vijaylata Pathania, Baljinder Singh Gill, Devinder Singh, Gopi Chand, Bikram Singh, Rakesh Deosharan Singh, Paramvir Singh Ahuja

**Affiliations:** Natural Product Chemistry and Process Development Division, Council of Scientific and Industrial Research-Institute of Himalayan Bioresource Technology (CSIR-IHBT), Post Box No. 6, Palampur, 176 061 HP India; Department of Agronomy, Punjab Agricultural University, Ludhiana, 141004 India; Regional Horticultural Research Station (RHRS), Dr YS Parmar University of Horticulture and Forestry, Jachh, Himachal Pradesh, India; Division of Hill Area Tea Science, CSIR-IHBT, Post Box No. 6, Palampur, 176 061 India; Division Biodiversity, CSIR-IHBT, Post Box No. 6, Palampur, 176 061 India; Division of Biotechnology, CSIR-IHBT, Post Box No. 6, Palampur, 176 061 India

**Keywords:** *Stevia rebaudiana*, Secondary metabolite, Crop ecology, Plant nutrition, Spatial variability, Cytokinin

## Abstract

**Background:**

Plant nutrition and climatic conditions play important roles on the growth and secondary metabolites of stevia (*Stevia rebaudiana* Bertoni); however, the nutritional dose is strongly governed by the soil properties and climatic conditions of the growing region. In northern India, the interactive effects of crop ecology and plant nutrition on yield and secondary metabolites of stevia are not yet properly understood. Thus, a field experiment comprising three levels of nitrogen, two levels of phosphorus and three levels of potassium was conducted at three locations to ascertain whether the spatial and nutritional variability would dominate the leaf yield and secondary metabolites profile of stevia.

**Results:**

Principal component analysis (PCA) indicates that the applications of 90 kg N, 40 kg P_2_O_5_ and 40 kg K_2_O ha^−1^ are the best nutritional conditions in terms of dry leaf yield for CSIR-IHBT (Council of Scientific and Industrial Research- Institute Himalayan Bioresource Technology) and RHRS (Regional Horticultural Research Station) conditions. The spatial variability also exerted considerable effect on the leaf yield and stevioside content in leaves. Among the three locations, CSIR-IHBT was found most suitable in case of dry leaf yield and secondary metabolites accumulation in leaves.

**Conclusions:**

The results suggest that dry leaf yield and accumulation of stevioside are controlled by the environmental factors and agronomic management; however, the accumulation of rebaudioside-A (Reb-A) is not much influenced by these two factors. Thus, leaf yield and secondary metabolite profiles of stevia can be improved through the selection of appropriate growing locations and proper nutrient management.

## Background

Stevia (*Stevia rebaudiana* Bertoni), a perennial herb of the Asteraceae family and native to South America (Paraguay and Brazil), is widely grown for its sweet leaf. Stevia is being commercially cultivated in Japan, China, Brazil, Paraguay, Mexico, Russia, Indonesia, Korea, USA, India, Tanzania, Canada and Argentina [1-3]. Though China is the largest stevia producer in the World market, Japan and Korea are the main consumers [4]. The worldwide researches in connection with stevia have mainly focused on the sweet-tasting diterpenoid steviol glycosides (SGs), which are used as a non-sucrose and non-caloric sweetener in a wide range of food products. In stevia, the SGs are mainly accumulated within its leaves, followed by stems, seeds and roots [5]. Amongst the known SGs, the most abundant glycoside in stevia leaf is stevioside, which is about 300 times sweeter than sucrose [6]. Rebaudioside-A (Reb-A), the second most abundant compound, is better suited than stevioside for use in foods and beverages due to its pleasant taste [7,8]. Thus there is a big challenge for agronomists and plant breeder to maintain the desirable level of Reb-A/ stevioside ratio in stevia leaves.

The worldwide demand for stevia is steadily increasing, since worldwide main regularity authorities (European Food Safety Authority, The US Food and Drug Administration, The Joint FAO/WHO Expert Committee on Food Additives, Food Standards Australia New Zealand) have approved the use of SGs, extracted from stevia leaves, as a dietary supplement [9-12]. To meet the burgeoning demand of stevia, it is imperative to increase the production through vertical as well as horizontal approaches. However, the understanding the growth behaviour, accumulation patterns of secondary metabolites and nutrient uptake dynamics in different agro-climatic conditions are prerequisite for introducing a new crop in a particular region.

The variability of SGs accumulation pattern in leaves during ontogeny of stevia is considerably influenced by the cultivar variations [5], photoperiod [13,14], temperature [15] and available nutrients [3,16]. It has also been reported that the leaf biomass and the concentration of active compounds depend upon the growing conditions and agronomic practices [17]. Among the agronomic practices, reliable nutrient supply is the most important factor for higher crop yield. Among the 17 essential plant nutrients, N, P and K are the most often limiting macronutrients for plant growth and development. Nitrogen is an essential element of key macromolecules such as proteins, nucleic acids, some lipids, and chlorophylls [18,19]. Phosphorus is also a component of nucleic acids, phospholipids, and ATP [20]. Potassium, third most essential macronutrient of plant, plays a central role in many fundamental metabolic processes, such as turgor driven movements, osmoregulation, control of membrane polarization and protein biosynthesis [21]. Thus, plants cannot perform properly without a reliable supply of these nutrients. Moreover, high dose fertilizer mainly N is harmful for soil health, especially when applied above the economic optimum dose.

The climatic factors are equally responsible for determining the vegetative growth and secondary metabolites of stevia. Stevia is an obligate short-day (SD) plant with a critical day length of about 12 h [22]. Under long-day (LD) condition, the vegetative growth phase of SD plant is retained for long time by prohibiting precocious flowering. It was reported that the LD conditions significantly increased leaf biomass and stevioside content in stevia leaves [13,23]. Therefore, the stevia plant should be grown under LD conditions to obtain greater leaf biomass with higher stevioside content. Nevertheless, under natural conditions, LD generally happens during the summer, and during this time other abiotic factors such as temperature and solar irradiance are generally not ideal for field production of stevia [23].

Thus, it is clear that standardization of nutritional doses particularly N, P and K for different agro-climatic conditions is essential for increasing the biomass yield and secondary metabolites of stevia. The sole and interaction effects of N, P and K on leaf yield and secondary metabolites of stevia have not been systematically investigated so far under different climatic conditions of northern India. The optimum doses of N, P and K for higher leaf yield under different agro-climatic conditions in India are not known. The synergistic and antagonistic effects of N, P, and K on stevia are also unknown. Thus, the objectives of this study were to (i) investigate the sole and interaction effects of N, P and K on yield, and the SGs’ accumulation in leaves; and (ii) standardize of N, P and K doses under different agro-climatic conditions.

## Methods

### Experimental location, climate and soil characteristics

**Figure 1:**
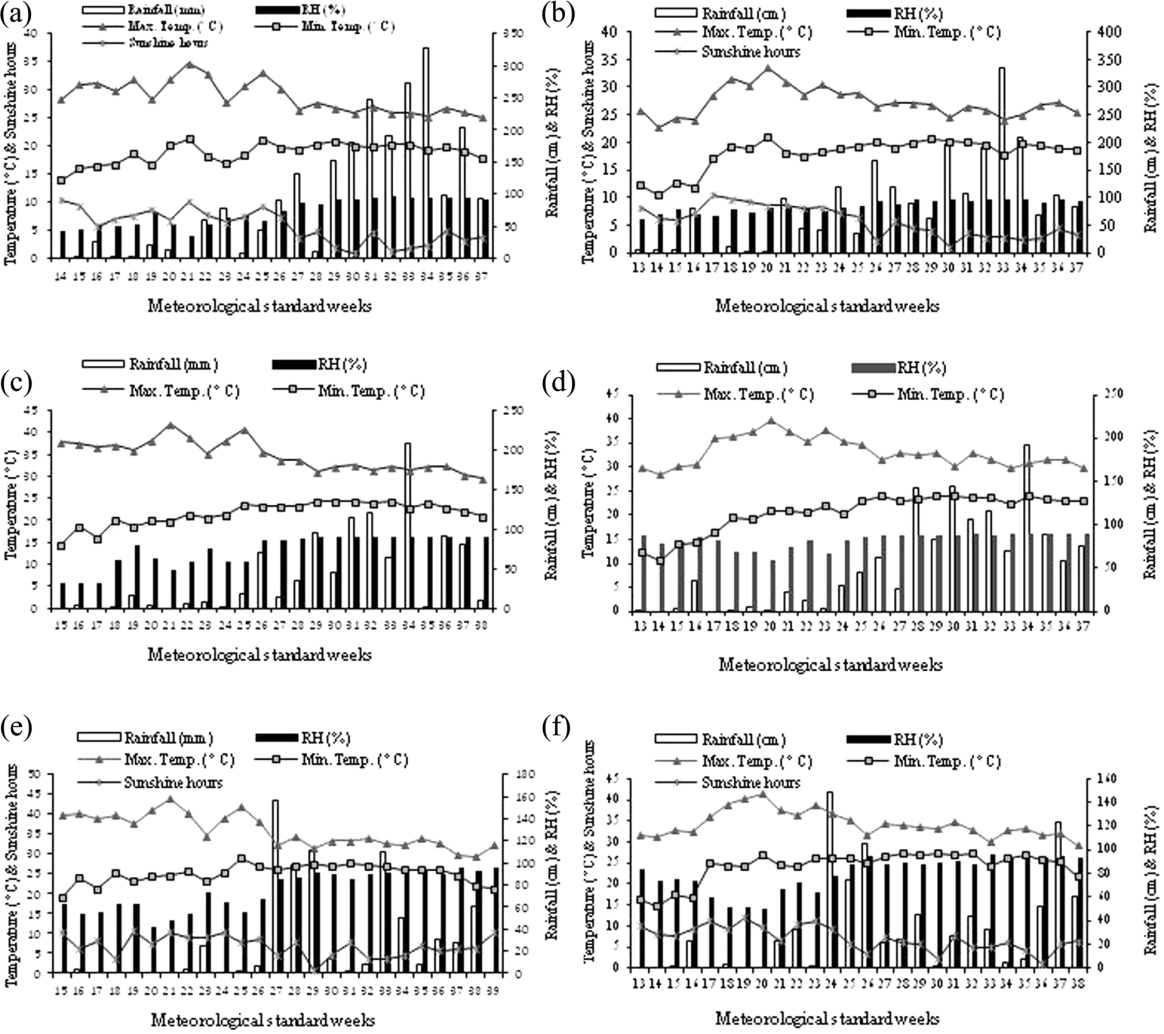
**Weekly mean maximum and minimum temperature (°C), sunshine hours (SS), rainfall (cm) and relative humidity (RH %) during the growing season of 2010 and 2011 at CSIR-IHBT (a, b), RHRS (c, d) and PAU (e, f).**

The investigations were carried out during 2010 and 2011 growing seasons, at three experimental locations. The sites were experimental farm of CSIR-Institute of Himalayan Bioresource Technology (CSIR-IHBT), Palampur; Regional Horticultural Research Station (RHRS), Jachh and Agronomy research farm of Punjab Agricultural University (PAU), Ludhiana. The sites were selected based on the variability of agro-climatic conditions and soil characteristics. According to the USDA soil taxonomy classification system the soils of Palampur, Jachh, and Ludhiana belong to Alfisols [24], Entisols [25], and Inceptisols [26], respectively. The details of geophysical situation, soil characteristics and weather conditions during the investigating years are presented in the Table 1 and Figure 1.

**Table 1:** **Physico-chemical properties of soil and Geophysical positioning of the experimental sites**

**Parameter**	**CSIR-IHBT**		**RHRS**		**PAU**	
	**2010**	**2011**	**2010**	**2011**	**2010**	**2011**
Soil type	Silty clay	Silty clay	Loamy sand	Loamy sand	Sandy loam	Sandy loam
pH (1:2.0)	6.40	5.70	7.50	7.42	7.82	7.74
Organic carbon (%)	1.98	1.36	0.70	0.75	0.26	0.30
Available nitrogen (g kg^−1^ soil)	0.136	0.147	0.118	0.116	0.54	0.61
Available phosphorus (g kg^−1^ soil)	0.027	0.025	0.006	0.007	0.009	0.008
Available potassium (g kg^−1^ soil)	0.207	0.205	0.080	0.083	0.107	0.109
Altitude (m from msl)	1393	1393	431	431	247	247
Longitude	32° 6′ 47″ N and 76° 33′ 46″ E	32° 6′ 47″ N and 76° 33′ 46″ E	32° 16′ N and 75° 51′	32° 16′ N and 75° 51′	30° 56′ N and 75° 52′ E	30° 56′ N and 75° 52′ E

### Plant material, application of treatments and crop management

The record of cropping scheme indicated that during 2009, the preceding year of field experimentation, stevia was grown for general purpose during spring season and remained fallow during winter. For transplanting the stevia seedlings the land was ploughed two times by power tiller to bring the good tilth of soil, and finally the land was leveled manually. Seventy-five-days-old stevia seedlings were transplanted at the end of 14th meteorological standard week (MSW) at Palampur in 2010, whereas at Jachh and Ludhiana the seedlings were transplanted at the starting of 15th MSW. In 2011, seedlings were transplanted during 13th MSW at all three locations. The planting geometry was a square shape with the space of 45 cm × 45 cm. The sizes of plots were 10 m^2^ (4 × 2.5 m). Forty five plants were accommodated in each plot. The experiment was laid out as three factors factorial arrangement in randomized block design (RBD) with three replications. Eighteen treatment combinations comprising three levels of N (N_1_ = 30 kg ha^−1^, N_2_ = 60 kg ha^−1^ and N_3_ = 90 kg ha^−1^), two levels of P (P_1_ = 20 kg P_2_O_5_ ha^−1^ and P_2_ = 40 kg P_2_O_5_ ha^−1^) and three levels of K (K_1_ = 20 kg K_2_O ha^−1^, K_2_ = 40 kg K_2_O ha^−1^ and K_3_ = 60 kg K_2_O ha^−1^) were tested. A half quantity of N and full quantity of P and K as per treatment were applied at the time of transplanting, while the remaining half quantity of N was applied into two equal doses at 30 and 60 days after transplanting (DAT). The N, P and K were applied through urea (46% N), single super phosphate (16% P_2_O_5_) and muriate of potash (60% K_2_O), respectively.

### Growth data and yield

For growth observation, two plants were randomly selected from centre of each plot then cut at 15 cm height from the ground level at 1st harvest (120 DAT), and both the plants were marked with an aluminum tag for the next observation during 2nd harvest (165 DAT). During second observation roots were removed from 0 to 25 cm soil layer for N, P and K analysis. After removal of plants from the field, leaves were separated from stem. Total number of branches (primary and secondary) per plant was quantified. The total area of fresh leaves under respective treatments was measured using a leaf-area meter (AM 300, ADC Bio-scientific Ltd., UK). Then the leaf area was expressed in the leaf area index (LAI). After recording the fresh weight of above-ground (during both harvest) and below-ground (only at 2nd harvest) parts, the samples were dried at 70 ± 2°C in an oven until a constant weight was attained to calculate the percentage of dry matter (DM) accumulation. These dry samples were also used for the estimation of N, P and K contents in different parts of the plant.

For determination of leaf and stem yield (fresh and dry), ten representative stevia plants from each plot were harvested at 15 cm height from the ground level during 1st harvest, whereas during 2nd harvest plants were cut at the ground level. Then the dry leaf and the stem yield from each plot were calculated by multiplying the fresh weight with factors, which are calculated from growth observation samples.

### Chlorophyll (Chl) determination

For the determination of chlorophyll (Chl), the leaves were collected from each experimental unit at the time of 1st harvesting at Palampur. The major veins were removed from the collected leaf samples to reduce the error. Then 200 mg fresh leaf sample was separated from each sample, and finally Chl was extracted in a solution of 80% acetone (v/v). Subsequently, the absorbances of the samples at 645 and 663 nm were recorded with a spectrophotometer (model T 90 + UV/vis, PG Instrument Ltd.). Finally, the fractions of Chl *a*, Chl *b* and total Chl (mg g^−1^ tissue) were estimated from the absorbance values as per standard equations recommended by Arnon [27].

### Determination of NPK in plant parts and soil analysis

Spatial and temporal dynamic of N, P and K uptake during the crop cycle were investigated lucidly for Palampur conditions. After recording growth data, representative samples of dry leaf, stem and root were prepared with a laboratory grinder having a sieve spacing of 0.7 mm to determine N, P and K partitioning in different parts. Prepared plant samples were digested with concentrated H_2_SO_4_ and selenium (Se) mixture as per the procedure suggested by Sahrawat et al. [28]. Total N was evaluated by micro-Kjeldahl method, while total P and K were estimated through a spectrophotometer (model T 90 + UV/vis, PG Instrument Ltd.) and a flame photometer (model BWB XP, BWB technologies UK Ltd., UK) respectively, according to Prasad et al. [29].

After harvesting, soil samples were collected from the surface layer (0–15 cm) for determination of pH, organic carbon (OC), available N (AN), available P (AP) and available K (AK). The pH of soil water suspension (1:2 w/v) was measured by pH meter (model Eutech Instruments pH 510), whereas the soil OC was determined by using the standard dichromate oxidation method of Nelson and Sommers [30]. Available N status of the soil was estimated after distilling the sample with alkaline potassium permanganate solution followed by titration [31]. Bray and Kurt P_1_ [32] method was used for estimation of available P, since the soil was acidic in nature. Available K in the soil was estimated by using earlier mentioned model of flame photometer after Mehlich-3 [33] extraction.

### Extraction and analysis of steviol glycosides

For estimation of steviol glycosides for all three locations, the leaves were collected from the middle portion of the plants from each plot at the time of harvest. The collected leaf samples were washed under running tap water to ensure the dust and microbes free samples. After removal of water from surface of the leaves, the samples were dried in a hot air oven at 40 ± 2°C until constant weight was attained. Then stevioside and Reb-A were determined with the help of Waters HPLC (996 Photodiode Array Detector) system. The extraction method and HPLC conditions were followed as described in our earlier paper [3]. The fractions of stevioside and Reb-A were quantified by the means of calibration curves, which were obtained from standard stevioside and Reb-A samples.

### Statistical analysis

All of the data obtained from three locations for 2 consecutive years were subjected to analysis of variance (ANOVA) using Statistica 7 software (Stat Soft Inc., Tulsa, Oklahoma, USA). The three-factors-factorial ANOVA was carried out separately for each year to estimate the variance components of main (N, P and K) effects and their reciprocal interactions (N × P, N × K, P × K and N × P × K) effects. Differences among the treatments were assessed with the least significant difference (LSD) only when the ANOVA *F*-test showed significance at *P* = 0.05. The data on secondary metabolites were presented as mean ± standard error (SE), and student paired *t*-test (*P* = 0.05) was applied to separate the treatment means. Principal component analysis (PCA) was also used to evaluate the nature of variation among the treatment combinations as a bi-plot. Factor loading values, which are presented as vectors, are the correlations of each variable (LAI, number of branches, leaf yield, stem yield, Chl and secondary metabolite profile) with the principal component (PC).

## Results

### Yield attributes

The analyzed data (Table 2) revealed that two main yield-attributes of stevia, number of branches (No. Plant^−1^) and LAI, were significantly affected by the level of N particularly at 1st harvest during both the years. During 1st harvesting stage, the maximum number of branches (7.58 and 11.86 No. plant^−1^) was registered with N_3_, that is significantly (*P* ≤ 0.05) different from N_1_, in both the experimental years, and from N_2_ in 2010. The effect of N_3_ and N_2_ on LAI at 1st harvest and total LAI were significantly higher compared with the effect of N_1_; however, these two treatments are statistically at par in both the years. The LAI at 2nd harvest was almost equal under all the treatments during both the years. At 1st harvest, the number of branches was significantly (*P* ≤ 0.05) affected by P during 2010, and highest number (6.64 No. plant^−1^) was recorded with P_2_. On the other hand, LAI at 1st harvest and total LAI were significantly (*P* ≤ 0.05) affected by the level of P, and the maximum LAI was recorded with P_2_ in both the years.

**Table 2:** **Effect of different levels N, P and K on yield attributes of stevia under CSIR-IHBT conditions**

**Treatment**	**Total branches (No plant** ^**−1**^ **)**				**Leaf area index(LAI)**						**Specific leaf weight(mg cm** ^**−2**^ **)**			
	**At 1st harvest**		**At 2nd harvest**		**At 1st harvest**		**At 2nd harvest**		**Total LAI**		**At 1st harvest**		**At 2nd harvest**	
	**2010**	**2011**	**2010**	**2011**	**2010**	**2011**	**2010**	**2011**	**2010**	**2011**	**2010**	**2011**	**2010**	**2011**
Nitrogen Level														
N_30_	5.29	9.06	18.29	23.5	1.41	1.67	0.22	0.2	1.63	1.88	7.47	7.54	7.64	9.33
N_60_	6.63	10.97	22.94	26.61	1.71	2.03	0.26	0.24	1.97	2.27	7.46	7.82	11.38	9.25
N_90_	7.58	11.86	25.34	27.72	1.77	2.12	0.27	0.25	2.04	2.37	7.43	8.39	14.75	9.97
SEm(±)	0.30	0.52	0.83	1.77	0.04	0.04	0.02	0.013	0.05	0.04	0.27	0.67	0.89	0.75
CD(P = 0.05)	0.86	1.50	2.38	NS	0.11	0.12	NS	NS	0.13	0.13	NS	NS	2.56	NS
Phosphorus Level														
P_20_	5.97	10.41	21.31	25.54	1.59	1.89	0.24	0.22	1.83	2.1	7.43	7.76	11.06	9.79
P_40_	7.03	10.85	23.07	26.35	1.68	2.00	0.26	0.24	1.91	2.25	7.59	8.09	11.45	9.24
SEm (±)	0.24	0.43	0.68	1.45	0.03	0.03	0.01	0.011	0.04	0.03	0.22	0.55	0.73	0.62
CD(P = 0.05)	0.70	NS	NS	NS	0.09	10	NS	NS	0.11	0.10	NS	NS	NS	NS
Potassium Level														
K_20_	6.4	10.06	20.29	25.28	1.56	1.82	0.22	0.23	1.78	2.05	7.19	7.39	11.13	9.15
K_40_	6.46	11.53	24.11	26.97	1.61	2.04	0.25	0.24	1.88	2.27	7.81	8.07	12.39	9.72
K_60_	6.64	10.31	22.18	25.58	1.73	1.98	0.27	0.22	1.99	2.02	7.36	8.3	10.25	9.68
SEm (±)	0.30	0.52	0.83	1.77	0.04	0.04	0.02	0.013	0.05	0.04	0.27	0.67	0.89	0.75
CD(P = 0.05)	NS	NS	2.38	NS	0.11	0.12	NS	NS	0.13	0.13	NS	NS	NS	NS
Interaction effect														
CD of N × P	NS	NS	NS	NS	NS	NS	NS	NS	NS	0.18	NS	NS	NS	NS
CD of N × K	NS	NS	2.35	NS	NS	NS	NS	NS	NS	0.22	NS	NS	NS	NS
CD of P × K	NS	NS	NS	NS	NS	NS	0.06	NS	NS	NS	NS	NS	3.61	NS
CD of N × P × K	NS	NS	NS	NS	NS	NS	NS	NS	NS	NS	NS	NS	NS	NS

N_1_, N_2_ and N_3_ are the level of nitrogen @ 30, 60 and 90 kg ha^−1^, respectively. P_1_ and P_2_ are the level of phosphorus (P_2_O_5_) @ 20 and 40 kg ha^−1^, respectively, while K_1_, K_2_ and K_3_ are representing the level of potassium (K_2_O) @ 20, 40 and 60 kg ha^−1^, respectively.

The effect of K on the number of branches was not significant (*P* ≥ 0.05) at 1st harvest; however, the maximum number of branches (6.64 and 11.53 No. plant-1) was recorded with K_2_ and K_3_ during 2010 and 2011, respectively. Among the K levels, the maximum LAI at 1st harvest and total LAI were recorded with K_3_ and K_2_ in 2010 and 2011, respectively, and these two treatments were significantly (*P* ≤ 0.05) different from K_1_. Though the SLW of stevia during 1st harvest was not significantly (*P* ≥ 0.05) influenced by the level of NPK doses, the marginal improvement of SLW was observed with the moderate level of N and K (N_2_ and K_2_) and higher level of P.

### Leaf yield, stem yield and harvest index (HI)

The data presented in Table 3 showed that the performance of stevia in terms of dry leaf yield (t ha^−1^) was superior under CSIR-IHBT conditions. Nevertheless, least performance was found under PAU conditions. The analyzed data (Table 3) also revealed that the overall effects of N, P and K on dry leaf yield (t ha^−1^) of stevia were significant (*P* ≤ 0.05) under CSIR-IHBT and RHRS conditions in 2010 and 2011. At PAU, dry leaf yield was not significantly affected by K (*P* ≥ 0.05) in both the years. Irrespective of P and K fertilization, the dry leaf yield (t ha^−1^) of stevia was increased with the corresponding increasing level of N at all 3 locations in both the years. Nevertheless, the magnitude of increase from N_1_ to N_2_ was higher compared with N_2_ to N_3_ particularly under CSIR-IHBT and PAU conditions. Under CSIR-IHBT conditions, N_3_ significantly (*P* ≤ 0.05) increased dry leaf yield (t ha^−1^) by about 36 and 42%, irrespective of P and K treatments, compared with N_1_ during 2010 and 2011, respectively. Similarly, significantly (*P* ≤ 0.05) higher dry leaf yield was also recorded with N_3_ compared with N_1_ under RHRS and PAU conditions in both the years. Moreover, the effect of climatic conditions was more pronounced on dry leaf yield (t ha^−1^). Irrespective of P and K treatments, the maximum dry leaf yield (1.69 and 1.91 t ha^−1^) of stevia which was recorded with 90 kg N ha^−1^ under CSIR-IHBT conditions, was about 62 and 164% higher at the same level of N compared with RHRS and PAU, respectively, on polled basis.

**Table 3:** **Effect of different levels N, P and K on yield (t ha** ^**−1**^ **) and harvest index (HI) of stevia under different experimental locations**

**Treatment**	**Dry leaf yield (t ha** ^**−1**^ **)**						**Dry stem yield (t ha** ^**−1**^ **)**						**Harvest Index (HI)**					
	**CSIR-IHBT**		**RHRS**		**PAU**		**CSIR-IHBT**		**RHRS**		**PAU**		**CSIR-IHBT**		**RHRS**		**PAU**	
	**2010**	**2011**	**2010**	**2011**	**2010**	**2011**	**2010**	**2011**	**2010**	**2011**	**2010**	**2011**	**2010**	**2011**	**2010**	**2011**	**2010**	**2011**
**Nitrogen Level**																		
**N**_**30**_	1.24	1.34	0.79	0.91	0.39	0.6	1.83	1.99	1.42	1.43	0.78	1.19	0.41	0.42	0.36	0.39	0.34	0.33
**N**_**60**_	1.56	1.69	0.93	1.04	0.52	0.81	2.19	2.48	1.47	1.56	0.76	1.47	0.42	0.41	0.39	0.4	0.4	0.35
**N**_**90**_	1.69	1.91	1.03	1.19	0.53	0.83	2.35	2.65	1.56	1.69	0.74	1.53	0.42	0.42	0.39	0.41	0.41	0.35
**SEm(±)**	0.04	0.03	0.03	0.02	0.02	0.04	0.04	0.05	0.03	0.03	0.03	0.08	0.007	0.004	0.009	0.007	0.007	0.011
**CD(P = 0.05)**	0.10	0.09	0.08	0.06	0.05	0.13	0.12	0.14	0.08	0.08	NS	0.22	NS	NS	0.025	NS	0.021	NS
**Phosphorus Level**																		
**P**_**20**_	1.44	1.55	0.87	1.01	0.47	0.68	1.94	2.22	1.48	1.55	0.73	1.35	0.42	0.41	0.37	0.39	0.39	0.34
**P**_**40**_	1.55	1.74	0.98	1.08	0.48	0.8	2.3	2.52	1.49	1.56	0.79	1.45	0.4	0.41	0.39	0.41	0.38	0.35
**SEm (±)**	0.03	0.03	0.02	0.02	0.01	0.04	0.03	0.04	0.02	0.02	0.03	0.06	0.006	0.003	0.007	0.006	0.006	0.009
**CD(P = 0.05)**	0.08	0.07	0.06	0.05	NS	0.11	0.10	0.12	NS	NS	NS	NS	0.016	NS	0.020	0.016	NS	NS
**Potassium Level**																		
**K**_**20**_	1.38	1.51	0.84	0.99	0.47	0.74	1.91	2.20	1.49	1.53	0.75	1.31	0.41	0.41	0.37	0.39	0.38	0.36
**K**_**40**_	1.62	1.74	0.98	1.08	0.49	0.76	2.11	2.44	1.52	1.59	0.77	1.48	0.43	0.42	0.39	0.4	0.38	0.34
**K**_**60**_	1.50	1.69	0.95	1.07	0.49	0.73	2.34	2.48	1.54	1.56	0.76	1.41	0.39	0.41	0.38	0.4	0.39	0.34
**SEm (±)**	0.04	0.03	0.03	0.02	0.02	0.04	0.04	0.05	0.03	0.03	0.03	0.08	0.007	0.004	0.009	0.007	0.007	0.011
**CD(P = 0.05)**	0.10	0.09	0.08	0.06	NS	NS	0.12	0.14	0.08	NS	NS	NS	0.02	NS	NS	NS	NS	NS
**Interaction effect**																		
**CD of N × P**	NS	NS	NS	NS	NS	NS	0.16	0.20	NS	NS	NS	NS	NS	NS	NS	NS	NS	NS
**CD of N × K**	0.18	0.16	NS	NS	NS	NS	0.20	NS	NS	NS	NS	NS	0.04	NS	NS	NS	NS	NS
**CD of P × K**	NS	NS	NS	NS	NS	NS	0.16	NS	NS	NS	NS	NS	0.03	NS	NS	NS	NS	NS
**CD of N × P × K**	NS	NS	NS	NS	NS	NS	NS	NS	NS	NS	NS	NS	NS	NS	NS	NS	NS	NS

N_1_, N_2_ and N_3_ are the level of nitrogen @ 30, 60 and 90 kg ha^−1^, respectively. P_1_ and P_2_ are the level of phosphorus (P_2_O_5_) @ 20 and 40 kg ha^−1^, respectively, while K_1_, K_2_ and K_3_ are representing the level of potassium (K_2_O) @ 20, 40 and 60 kg ha^−1^, respectively.

The dry leaf yield in response to P was significant (*P* ≤ 0.05) under CSIR-IHBT and RHRS conditions and the maximum yield (Table 3) was recorded with P_2_ in both the years. However, the effect of P in terms of dry leaf yield was not significant (*P* ≥ 0.05) at PAU in 2010. Irrespective of N and P application, the dry leaf yield (t ha^−1^) of stevia was significantly (*P* ≤ 0.05) affected by different levels of K fertilization under CSIR-IHBT and RHRS conditions in both the years. The maximum dry leaf yields of stevia under CSIR-IHBT conditions were 1.62 and 1.74 t ha^−1^ during 2010 and 2011, respectively, with 40 kg K ha^−1^. However, further increases in K application resulted in a decline in dry leaf yield, and the lowest value (1.38 and 1.51 t ha^−1^) was observed with the application of 20 kg K ha^−1^. Among the 1st order interactions (N × P, N × K, and P × K), the effect of N × K on dry leaf yield was significant under CSIR-IHBT conditions, however, the 2nd order (N × P × K) interaction effects were insignificant (*P* ≥ 0.05) at all 3 locations (Table 3).

The analyzed data (Table 3) revealed that the effect of applied N on dry stem yield (t ha^−1^) was significant (*P* ≤ 0.05) under CSIR-IHBT and RHRS conditions in both the years. The trend of stem yield was similar to leaf yield, and the maximum stem yield (2.35 and 2.65 t ha^−1^) was recorded with N_3_ under CSIR-IHBT conditions in both the years. Though the effects of P and K were negligible under RHRS and PAU conditions, the significant effects were found at CSIR-IHBT. The data revealed (Table 3) that the harvest index (HI) of stevia was not markedly influenced by different levels of N, P and K under all 3 conditions. However, the application of 90 kg N ha^−1^ resulted in significantly (*P* ≤ 0.05) higher HI compared with 30 kg N ha^−1^ under RHRS and PAU conditions during 2010.

### Physical and economical optimal dose (kg ha^−1^)

The physical and economical optima of N and K fertilizer doses were estimated for IHBT and PAU conditions by derivation of quadratic equations, which are presented in Table 4 for the respective sites. The physical optima of N were 106.67 and 74.44 kg ha^−1^ for CSIR-IHBT and PAU conditions, respectively. However, the physical optima of K were estimated for all 3 locations, since the yield responses were quadratic. The physical optima of K for CSIR-IHBT, RHRS and PAU conditions were 44.62, 39.75 and 45.00 kg ha^−1^, respectively. Economical optima of N and K were estimated based on prevailing market price of urea (Rs. 5.50 kg^−1^), muriate of potash (Rs. 12.00 kg^−1^) and dry leaf of stevia (Rs. 130.00 kg^−1^) in India. Economical optima of N were very close to physical optima, which are 106.16 and 73.93 kg ha^−1^ for CSIR-IHBT and PAU conditions, respectively. The economical optima of K for CSIR-IHBT, RHRS and PAU conditions were 44.43, 39.37 and 43.08 kg ha^−1^, respectively.

**Table 4:** **Predictive regression equations and physical and economical optimal doses of N and K under different agro-climatic conditions**

**Experimental site**	**Plant nutrient**	**Regression equation**	**Physical optima (kg ha** ^**−1**^ **)**	**Economical optima (kg ha** ^**−1**^ **)**
CSIR-IHBT	Nitrogen	y = 0.795 + 0.0192^*^x − 0.00009^*^x^2^	106.67	106.16
	Potassium	y = 0.89 + 0.0358^*^x − 0.0004^*^ x^2^	44.62	44.43
RHRS	Nitrogen	y = 0.705+ 0.005^*^x − 0.000006^*^ x^2^	-	-
	Potassium	y = 0.665 + 0.0159^*^x − 0.0002^*^ x^2^	39.75	39.37
PAU	Nitrogen	y = 0.17+ 0.0134^*^x − 0.00009^*^ x^2^	74.44	73.93
	Potassium	y = 0.55 + 0.0036^*^x − 0.00004^*^ x^2^	45.00	43.08

*mark indicates that the corresponding values are significant at *P* = 0.05 . The physical and economical optima of N for RHRS were not calculated since the relation between N and dry leaf yield (t ha^−1^) was almost linear.

### Regression and correlation analysis

The correlation analysis revealed that dry leaf yield (t ha^−1^) was significantly (*P* ≤ 0.05) and positively correlated with total LAI with correlation coefficients of 0.83 in 2010 and 0.77 in 2011. A significant (*P* ≤ 0.05) positive correlation was also found with the number of branches at 1st harvest having correlation coefficients of 0.77 and 0.90 during 2010 and 2011, respectively. The regression between yield and yield attributes is explained by the equation of

$$ \widehat{\mathrm{Y}}=-1.2338+0.0242{\mathrm{X}}_1-0.0037{\mathrm{X}}_2+0.6974**{\mathrm{X}}_3+0.1325**{\mathrm{X}}_4+0.0241**{\mathrm{X}}_5\left({\mathrm{R}}^2 = 0.973**\right) $$

Where Ŷ is the dry leaf yield (t ha^−1^), X_1_ the number of branches per plant at 1st harvest, X_2_ the number of branches per plant at 2nd harvest, X_3_ the total LAI, X_4_ the SLW at 1st harvest, and X_5_ is the SLW at 2nd harvest. The R^2^ values indicated that more than 97% of the variability of dry leaf yield (t ha^−1^) was explained by these variables. The regression coefficients of total LAI, SLW at 1st harvest and SLW at 2nd harvest were also significant (P ≤ 0.01).

### Spatial and temporal nutrient dynamic in plant

**Figure 2:**
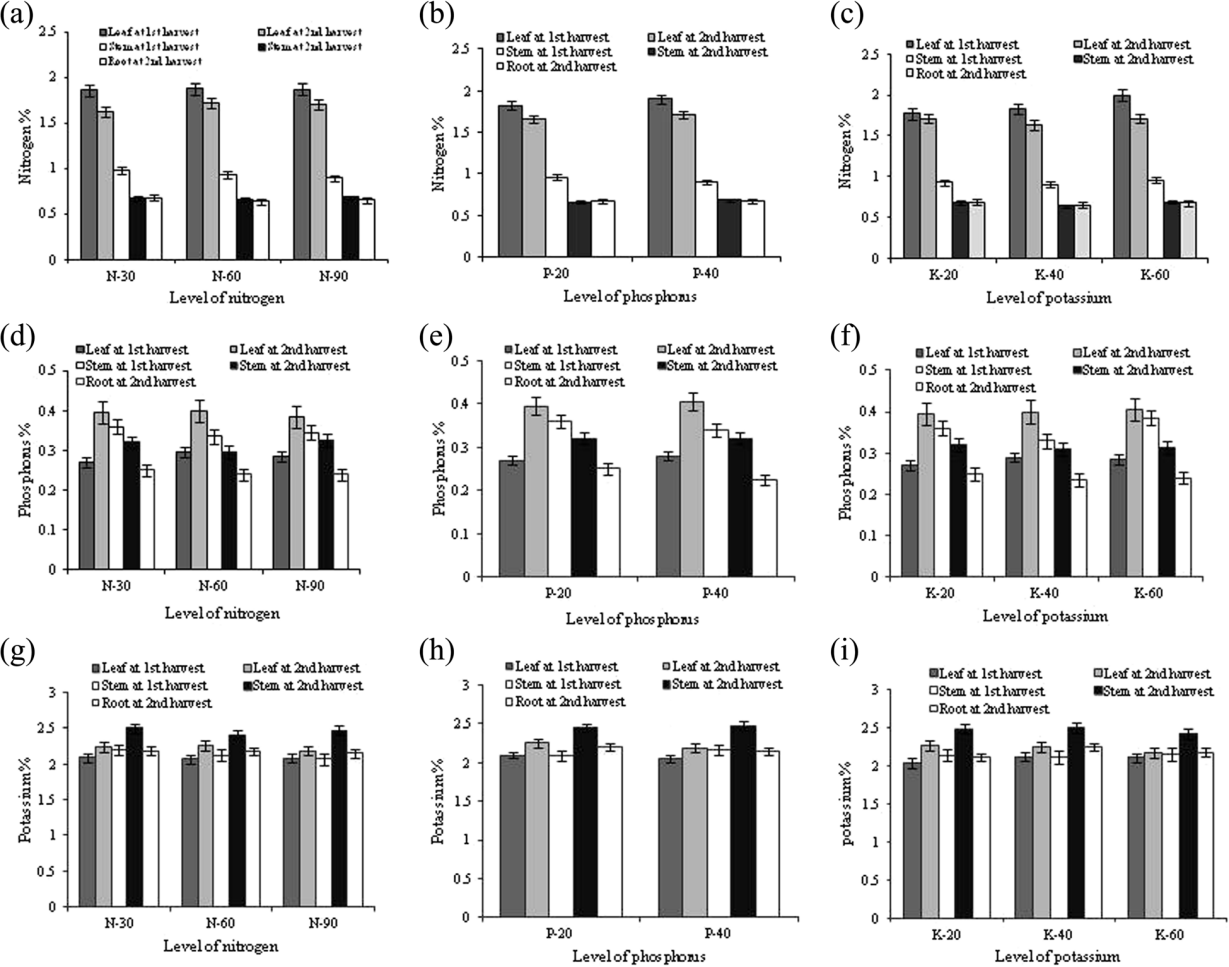
**Spatial and temporal accumulation of N (a-c), P (d-f) and K (g-i) in stevia plant as influenced by applied N, P and K at CSIR-IHBT.** The mean values of two years pooled data are presented. Vertical bars indicate a mean standard error (±).

Spatial and temporal nutrient (N, P and K) dynamics of stevia under CSIR-IHBT conditions are illustrated in the Figure 2. The overall NPK accumulation patterns in response to different levels of N, P and K were insignificant (*P* ≥ 0.05). However, irrespective of nutritional treatment, the considerable differences were found due to spatial and temporal variations. The highest quantity of N was accumulated in the leaf followed by stem and root, and the magnitude of accumulation during 1st harvest was marginally higher compared with 2nd harvest. However, the trend of N accumulation in the leaf was similar at both harvesting stages, and the highest magnitude was recorded with N_2_ (1.88 and 1.72 %), P_2_ (1.91 and 1.71%) and K_3_ (2.0 and 1.71%) in the respective factors. The trend of N accumulation in the stem in two harvesting stages was not similar under different nutritional treatments. In contrast to N, the accumulation of P in leaf was marginally higher at 2nd harvest. Similarly, K content in leaf and stem was higher during 2nd harvesting. However, the effects of applied K in terms of K content (%) in leaf, stem and root were inconsistent.

### Chlorophyll (Chl) content in leaf

**Figure 3:**
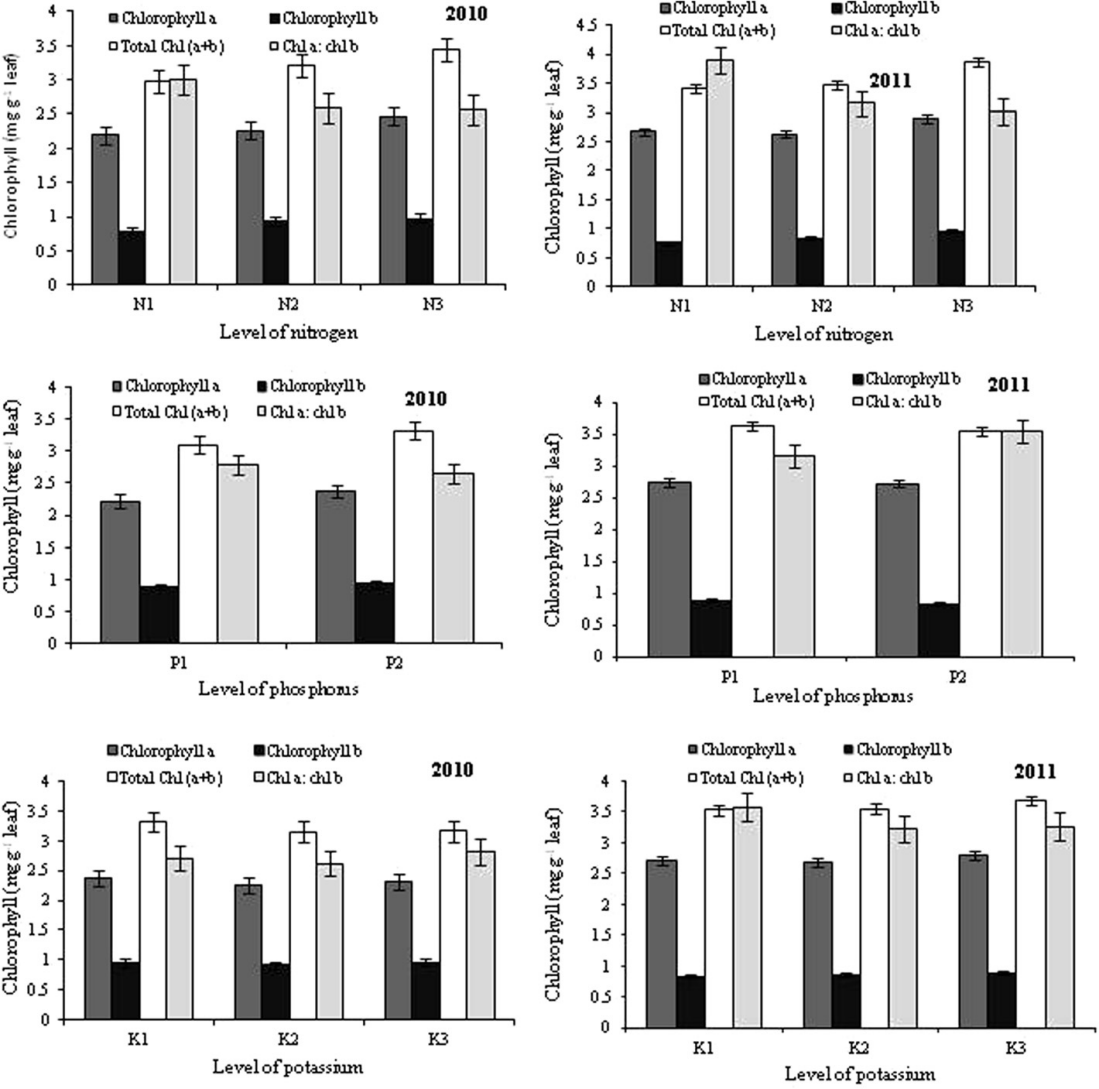
**Photosynthetic pigments in leaves of stevia plants grown under different levels of N, P and K at CSIR-IHBT.** The data represent the mean of two years. Vertical bars indicate a mean standard error (±).

The results presented in the Figure 3 showed that the effects of N, P and K on Chl *a* and Chl *b* were not significant (*P* ≥ 0.05) during 2010; however, the application of higher dose of N (90 kg ha^−1^) significantly increased Chl *b* content compared with low and moderate levels of N during 2011. Regardless of P and K, the total Chl content in leaves was also significantly (*P* ≤ 0.05) influenced by level of N during 2010 and 2011(Figure 3), and the utmost (3.45 and 3.86 mg g ^−1^) and least (2.98 and 3.42 mg g ^−1^) quantity were recorded with N_3_ and N_1_, respectively. Irrespective of P and K fertilization, the correlation between applied N and total Chl content was significant, with correlation coefficient of 0.99 (*P* ≤ 0.05) in 2010. On the other hand, P and K did not significantly (*P* ≥ 0.05) influence total Chl content.

### Secondary metabolites accumulation in leaf

The two major SGs in stevia leaf, stevioside and Reb-A, which were quantified for all 3 locations, are presented in Table 5. In this study, the overall effects of N, P and K on stevioside and Reb-A were not considerable under RHRS and PAU conditions. Nevertheless, the effect of N on stevioside and total SGs (stevioside + Reb-A) was significant (*P* ≤ 0.05) under CSIR-IHBT conditions, and the maximum quantity (12.68 and 16.2%) was recorded with the application of moderate quantity of N (60 kg ha^−1^). This treatment recorded about 27 and 18 % higher stevioside content in leaf, irrespective of P and K treatments, compared with N_1_ and N_3_, respectively. At PAU, the trend of stevioside accumulation under N treatments was similar to CSIR-IHBT conditions. Whereas, at RHRS, the total SGs content gradually increased with the application up to 90 kg N ha^−1^ but statistically at par (*P* ≥ 0.05) with the rest of N treatments. In addition, it was clear that the variations in stevioside accumulation in leaf at different locations were quite high compared with Reb-A (Table 5). Irrespective of nutritional treatments, overall performance in terms of secondary metabolites accumulation was better under CSIR-IHBT conditions compared with rest of the locations. In contrast to total SGs, the Reb-A content under PAU condition was similar to CSIR-IHBT. The least performance was found under RHRS conditions.

**Table 5:** **Comparison of secondary metabolite profile changes as influenced by applied N, P and K under different agro-climatic conditions**

**Treatment**	**Stevioside (ST) content (%)**			**Rebaudioside -A (Rab-A) content (%)**			**ST + Reb-A content (%)**			**ST: Reb-A**		
	**IHBT**	**RHRS**	**PAU**	**IHBT**	**RHRS**	**PAU**	**IHBT**	**RHRS**	**PAU**	**IHBT**	**RHRS**	**PAU**
Nitrogen Level												
N_30_	9.98 ± 0.69 a	5.42 ± 0.92 a	7.21 ± 0.22 a	3.38 ± 0.22 a	2.93 ± 0.29 a	4.48 ± 0.24 a	13.37 ± 0.75 a	8.35 ± 1.11a	11.70 ± 0.40 a	3.01 ± 0.26 a	1.82 ± 0.23 a	1.63 ± 0.08 a
N_60_	12.68 ± 0.43 b	6.23 ± 1.13 a	8.37 ± 1.02 a	3.52 ± 0.25 a	2.37 ± 0.43 a	3.30 ± 0.48 a	16.20 ± 0.43 b	8.6 0 ± 0.92 a	11.67 ± 0.88 a	3.75 ± 0.42 a	3.33 ± 0.98 a	3.04 ± 0.75 b
N_90_	10.73 ± 0.69 ab	7.15 ± 0.60 a	7.00 ± 1.013 a	3.42 ± 0.30 a	2.63 ± 0.37 a	3.67 ± 0.44 a	14.15 ± 0.88 ab	9.78 ± 0.57 a	10.67 ± 1.08 a	3.24 ± 0.26 a	3.00 ± 0.46 a	2.16 ± 0.60 ab
Phosphorus Level												
P_20_	10.99 ± 0.67 a	6.20 ± 0.74 a	7.51 ± 0.41 a	3.46 ± 0.19 a	2.99 ± 0.28 a	3.84 ± 0.41 a	14.44 ± 0.77 a	9.19 ± 0.76 a	11.36 ± 0.35 a	3.24 ± 0.21 a	2.25 ± 0.37 a	2.41 ± 0.55 a
P_40_	11.28 ± 0.58 a	6.33 ± 0.78 a	7.54 ± 0.90 a	3.42 ± 0.22 a	2.3 ± 0.27 a	3.79 ± 0.31 a	14.70 ± 0.62 a	8.63 ± 0.71 a	11.33 ± 0.90 a	3.42 ± 0.33 a	3.18 ± 0.65 a	2.14 ± 0.42 a
Potassium Level												
K_20_	11.78 ± 0.56 a	5.58 ± 0.69 a	7.65 ± 0.87 a	3.50 ± 0.87 a	2.60 ± 0.31 a	3.27 ± 0.59 a	15.28 ± 0.62 a	8.19 ± 0.72 a	10.92 ± 0.92 a	3.40 ± 0.20 a	2.30 ± 0.40 a	2.93 ± 0.78 a
K_40_	11.35 ± 0.36 a	6.78 ± 1.04 a	7.58 ± 0.24 a	3.7 0 ± 0.24 a	2.57 ± 0.41 a	4.37 ± 0.18 a	15.05 ± 0.43 a	9.35 ± 0.86 a	11.95 ± 0.30 a	3.08 ± 0.12 a	3.16 ± 0.81 a	1.75 ± 0.09 a
K_60_	10.27 ± 1.10 a	6.43 ± 1.035 a	7.35 ± 1.23 a	3.12 ± 1.23 a	2.77 ± 0.40 a	3.82 ± 0.36 a	13.38 ± 1.17 a	9.20 ± 1.12 a	11.17 ± 1.09 a	3.51 ± 0.54 a	2.68 ± 0.77 a	2.14 ± 0.63 a

The data are means ± SE (n = 6 for nitrogen; n = 9 phosphorus; n = 6 for potassium). Values with the same letter are not significantly different (*P* = 0.05) in the respective factors. N_1_, N_2_ and N_3_ are the level of nitrogen @ 30, 60 and 90 kg ha^−1^, respectively. P_1_ and P_2_ are the level of phosphorus (P_2_O_5_) @ 20 and 40 kg ha^−1^, respectively, while K_1_, K_2_ and K_3_ are representing the level of potassium (K_2_O) @ 20, 40 and 60 kg ha^−1^, respectively.

### Principal component analysis

**Figure 4:**
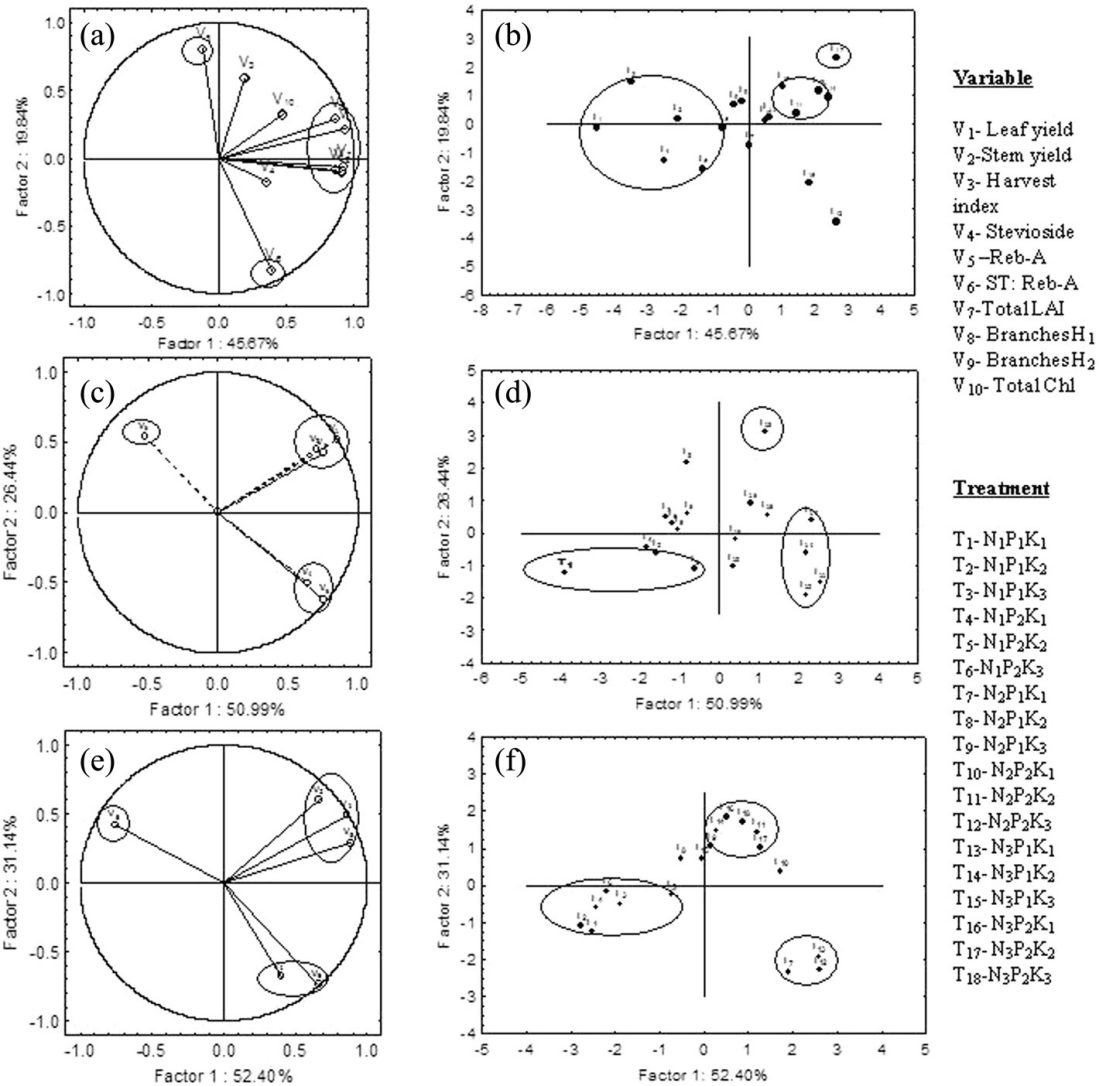
**Bi-plot of principal components based on mean value of yield, yield attributes secondary metabolites profile and Chl data.** Factor 1 and Factor 2 explain 65.51, 77.43 and 83.54 % of the data variation for CSIR-IHBT, RHRS and PAU, respectively. Figure **a**, **c** and **e** are the variable vector distributions; Figure **b**, **d** and **f** are the case distributions (treatment combinations). The loading values of variables are presented (a, c and e) as vectors in the space of the PCA bi-plots. N_1_, N_2_ and N_3_ are the level of nitrogen @ 30, 60 and 90 kg ha^−1^, respectively. P_1_ and P_2_ are the level of phosphorus @ 20 and 40 kg ha^−1^, respectively, while K_1_, K_2_ and K_3_ are representing the level of potassium @ 20, 40 and 60 kg ha^−1^, respectively.

Principal component analysis (PCA) was carried out using the set of 10 variables for CSIR-IHBT and 6 variables for RHRS and PAU conditions. The data presented in the Figure 4a-f revealed that the first two components, PC_1_ and PC_2_, explained 65.51, 77.43 and 83.54 % of the total variations for CSIR-IHBT, RHRS and PAU conditions, respectively. Figure 4a, c and e show the relationships among the variables in the space of the first two components (PC_1_ and PC_2_), and also indicate the magnitude of variable-contribution to the principal components for the respective locations. Under CSIR-IHBT condition, except Reb-A (V_5_), all variables [(leaf yield (V_1_), stem yield (V_2_), HI (V_3_), stevioside (V_4_), stevioside: Reb-A (V_6_), total LAI (V_7_), branches at 1st harvest (V_8_), branches at 2nd harvest (V_9_)and total Chl (V_10_)] are located in the positive coordinate of PC_1_. However, the loading values (correlation coefficient) of V_1,_ V_2_, V_7_, V_8_ and V_9_ with PC_1_ were too high (more than 0.8). The PCA bi-plot (Figure 4b.) separated the treatment T_17_ (N_3_P_2_K_2_) by PC_1_ and PC_2_ and placed in the positive coordinate of both PCs; whereas, the first 6 treatments (T_1_-T_6_) are located in the same cluster. The PCA bi-plots (Figure 4a and b) explained strong associations among the major variables for T_17_, and also confirming the data presented in the Tables 2 and 3.

The PCA bi-plots (Figure 4c and e) show a similar pattern of variable vectors distribution for RHRS and PAU conditions. It is clear that the PC_1_ has positive coefficients with 5 variables (V_1_, V_2_, V_3_, V_4_ and V_6_) and negative coefficients with V_5_. The V_1_, V_2_, V_3_ and V_6_ have similar heavy loadings for PC_1_. In case-distribution-plot (Figure 4d), T_17_ and T_18_ are separated along with PC_1_ and PC_2_, respectively, from rest of the treatments under RHRS conditions. Thus, the overall PCA output indicates that T_17_ (N_3_P_2_K_2_) represents the best nutritional conditions in terms of dry leaf (t ha^−1^) for CSIR-IHBT and RHRS. However, under PAU condition, there was no single treatment, which was distinctly different from rest of the treatments.

### Nutrient (NPK) uptake

**Figure 5:**
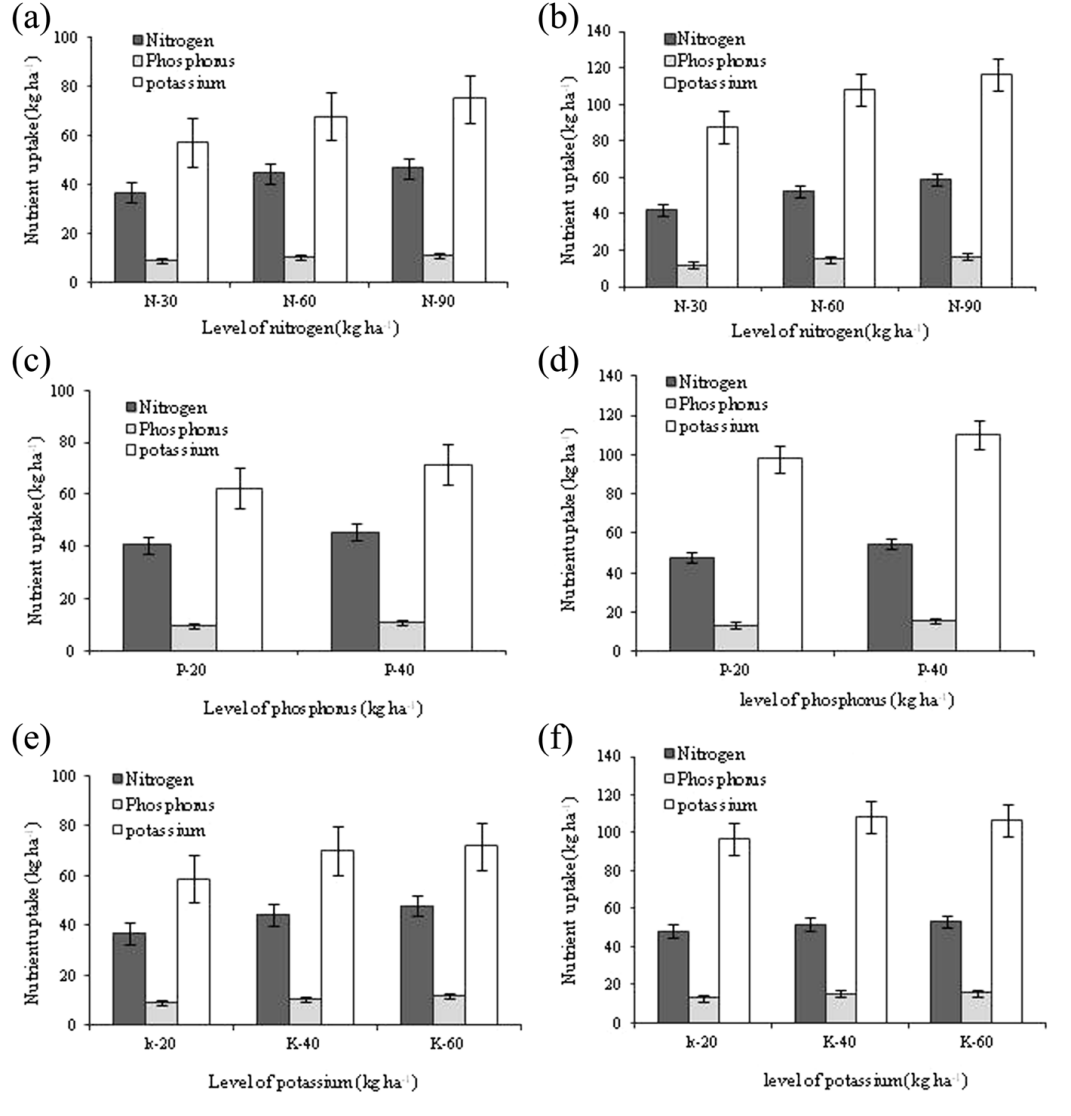
**Relative uptake of total N, P and K by above ground biomass of stevia under varying levels of N (a, b), P (c, d) and K (e, f) application at CSIR-IHBT.** Vertical bars indicate a mean standard error (±).

The data presented in the Figure 5 revealed that the nutrient (NPK) uptake by stevia (above ground parts) in response to different levels of N, P and K fertilizer was significant (*P* ≤ 0.05). Among the N levels, the application of higher dose (90 kg N ha^−1^) resulted in significantly higher N (47.02 and 59.05 kg ha^−1^), P (11.43 and 16.74 kg ha^−1^), and K (75.35 and 116.1 kg ha^−1^) uptake by stevia compared with lower dose (30 kg ha^−1^) in 2010 and 2011. Uptake of P and K also followed a trend similar to that observation for N, with greatest value was observed in plants, which received 90 kg N ha^−1^ in both the years. The effect of P on nutrient (NPK) uptake was significant (*P* ≤ 0.05), and the maximum values were recorded with 40 kg P ha^−1^ in both the years (Figure 5). We also observed that the application of higher dose of K (60 kg ha^−1^) significantly (*P* ≤ 0.05) increased N and P uptake in both the years and K uptake in 2010 compared with lower dose (30 kg ha^−1^).

### Chemical properties of soil after harvest

The chemical properties (pH, OC, AN, AP and AK) of the soil were not significantly (*P* ≥ 0.05) changed by the levels of NPK application except pH value in 2010 (Table 6). Significantly (*P* ≤ 0.05) lowest pH value (6.26) was registered with N_3_ compared with N_1_ in 2010. Soil OC was not significantly (*P* ≥ 0.05) influenced by the applied N; however, N_3_ maintained the highest value (2.57 and 2.20 %), and lowest (2.40 and 2.02 %) value was observed with N_2_ in both the years. In this study, the change of AN, AP and AK content in the soil was not considerable; however, maximum AN (222.83 and 327.88 kg ha^−1^) was recorded with N_1_. On the other hand, K content in the soil was marginally improved by the moderate level of K (40 kg ha^−1^) compared with lower (20 kg ha^−1^) and higher (60 kg ha^−1^) dose.

**Table 6:** **Effect of applied N P and K on soil pH, organic carbon (OC), available nitrogen (AN), available phosphorus (AP) and available potassium (AK) at CSIR-IHBT**

**Treatment**	**pH**		**OC (%)**		**AN (kg ha** ^**−1**^ **)**		**AP (kg ha** ^**−1**^ **)**		**AK (kg ha** ^**−1**^ **)**	
	**2010**	**2011**	**2010**	**2011**	**2010**	**2011**	**2010**	**2011**	**2010**	**2011**
Nitrogen Level										
N_30_	6.59	5.88	2.41	2.15	222.83	327.88	62.51	59.42	549.55	486.38
N_60_	6.35	5.85	2.40	2.02	212.72	319.35	67.65	55.12	557.75	494.15
N_90_	6.26	5.83	2.57	2.20	205.06	310.11	64.67	66.34	544.66	504.70
SEm(±)	0.08	0.04	0.12	0.10	7.87	12.08	4.33	7.42	10.02	19.85
CD(P = 0.05)	0.22	NS	NS	NS	NS	NS	NS	NS	NS	NS
Phosphorus Level										
P_20_	6.42	5.86	2.5	2.07	210.57	326.84	65.08	61.33	540.33	495.35
P_40_	6.39	5.85	2.43	2.18	216.50	311.39	64.81	59.26	560.98	494.81
SEm (±)	0.06	0.03	0.10	0.08	6.43	9.86	3.53	6.06	8.18	16.20
CD(P = 0.05)	NS	NS	NS	NS	NS	NS	NS	NS	NS	NS
Potassium Level										
K_20_	6.47	5.86	2.56	2.01	215.69	327.01	67.13	52.07	548.45	478.68
K_40_	6.42	5.82	2.37	2.25	208.89	314.29	57.99	67.85	559.35	503.53
K_60_	6.33	5.89	2.46	2.11	216.03	316.03	69.7	60.96	544.17	503.02
SEm (±)	0.08	0.04	0.12	0.10	7.87	12.08	4.33	7.42	10.02	19.85
CD(P = 0.05)	NS	NS	NS	NS	NS	NS	NS	NS	NS	NS
Interaction effect										
CD of N × P	NS	NS	NS	NS	NS	NS	NS	NS	NS	NS
CD of N × K	NS	NS	NS	NS	NS	NS	NS	NS	NS	NS
CD of P × K	0.31	NS	NS	0.47	NS	49.08	NS	NS	NS	NS
CD of N × P × K	NS	NS	NS	NS	NS	NS	NS	NS	NS	NS

N_1_, N_2_ and N_3_ are the level of nitrogen @ 30, 60 and 90 kg ha^−1^, respectively. P_1_ and P_2_ are the level of phosphorus (P_2_O_5_) @ 20 and 40 kg ha^−1^, respectively, while K_1_, K_2_ and K_3_ are representing the level of potassium (K_2_O) @ 20, 40 and 60 kg ha^−1^, respectively.

## Discussion

Branches and LAI, the main yield-attributes of stevia, were significantly (*P* ≤ 0.05) higher, particularly at 1st harvesting stage, with higher dose of nitrogen. Fagerstrom and Lohm [34] and Marschner [35] reported that N stimulated the leaf production probably due to the increasing production of cytokinin in root tips and their eventual export to the shoot. On one hand, NO_3_^−^ promotes lateral roots elongation through the accumulation of auxin [36]. On the other hand, NO_3_^−^ induces cytokinin production [37,38], which is necessary to encourage lateral root development in response to a systemic-N signaling [39]. It has also been reported that the foliar application of different NO_3_^−^ and NH_4_^+^ salts [(KNO_3_, Ca(NO_3_)_2_ and (NH_4_)_6_Mo_7_O_24_)] increased the number of branches and LAI compared with water spray control [3]. In the present study, the increase in LAI in response to increase in P level was probably due to enhanced availability of P, which improved leaf expansion and photosynthesis per unit leaf area. Maximum LAI was observed with high and moderate levels of K in 2010 and 2011, respectively. This result might be attributed to a longer leaf lifespan, which ultimately enhanced LAI. A positive effect of K fertilization on the leaf lifespan of field-grown almond tree was also reported [40].

In this study, the dry leaf yield (t ha^−1^) of stevia was increased by increasing the N level at all three locations. These results may be due to the fact that higher dose of N increased the availability of N in soil, and subsequently induced cytokinin synthesis in root tips and maintained desirable cytokinin and auxin ratio. Therefore, the maximum leaf yield was obtained with higher dose of N as a result of higher LAI. Ioio et al. [41] reported that root cell division and differentiation are controlled by the cytokinin and auxin ratio. Moreover, during embryogenesis, cytokinin and auxin control the events of major cell specification [42]. It has also been reported that limited supply of N decreased root growth, inhibited lateral root initiation, increased the C/N ratio within the plant, decreased photosynthesis, and early leaf senescence [43-47].

In the present study, the application of the higher dose of P (40 kg P_2_O_5_ ha^−1^) leads to considerably higher dry leaf yield compared with the lower dose of P (20 kg P_2_O_5_ ha^−1^). These results may be due to the fact that P is an essential component of key molecules such as nucleic acids, phospholipids, and ATP [20], which are necessary for photosynthesis, energy transfer, carbohydrate and protein synthesis, and lipid metabolism [48]. The moderate level of K was most effective in terms of dry leaf yield of stevia at all three locations. The results are in accordance with the findings of Laclaun et al. [49].

From the present study it is confirmed that the growth and dry matter accumulation of stevia are markedly governed by the prevailing environmental conditions during plantation and vegetative growth phases. The plants, grown under CSIR-IHBT conditions, produced maximum dry leaf and stem yield, while least performance was found under PAU condition. These results could be due to the fact that environmental conditions, particularly temperature was not favourable during plantation and vegetative growth phases at PAU. Sometime the maximum temperature at PAU reached more than 42°C during plant establishment and vegetative growth stages (Figure 1). The extremely high temperature and corresponding lower RH could have reduced photosynthetic activities, and lowered the yield at PAU.

The total Chl was significantly (P ≤ 0.05) increased with higher level of N. These results may be due to the fact that N is an essential component of green pigment of plants [50]. On the other hand, the Chl *a/b* ratio was lowest with higher dose of N. Hikosaka and Terashima [51] reported that Chl *a/b* ratio was decreased with the increase in N availability at a defined light intensity.

In this study, applied N, P and K had little effect in altering the concentration of N, P and K in plant body. This result was probably due to the dilution affect of nutrient content. The uptake of N, P and K (kg ha^−1^) by above ground biomass of stevia was increased progressively with the increase of N level from 30 to 90 kg ha^−1^. The better availability of N encourages root proliferation through auxin and cytokinin synthesis [36-38], resulting in removing more nutrients from large area and greater soil depth. The increased biomass production coupled with moderate concentration of N, P and K in leaf and stem also may be the cause of higher uptake of N, P and K under higher dose of N. Though the concentrations of P in leaf and stem were not changed significantly (*P* ≥ 0.05), the uptake of P was increased significantly (*P* ≤ 0.05) with higher dose of P. This increase was generally caused by higher dry leaf and stem yield. Mollier and Pellerin [52] reported that root growth of maize (*Zea mays* L.) was strongly reduced after a few days of P starvation, and the emergence of new axile roots and elongation of first-order lateral roots were also radically reduced. It has also been reported that P deficiency reduced absolute root growth of rice (*Oryza sativa* L.), and this reduction was more pronounced in genotypes with a low tolerance to P deficiency [53].

Stevioside accumulation in leaf was significantly improved by the moderate level of N under CSIR-IHBT conditions. This result might be attributed to synergistic effect with other essential nutrients, which improved the biochemical activities for increasing stevioside. The higher stevioside content in leaf with moderate level of N might be attributed to the desired level of photosynthetic pigments. Ladygin et al. [54] reported that accumulation of steviol glycosides in cells of stevia *in vivo* and *in vitro* was related to the extent of the development of the membrane system of chloroplasts and the content of photosynthetic pigments. The variation in stevioside accumulation in leaves due to location variation was quite high compared with Reb-A. Thus the results suggest that accumulation of stevioside is influenced by environmental and soil conditions. It has been reported that stevioside levels vary depending on the growing conditions and genotype [55]. In our study, Reb-A content did not much vary due to site variation, which suggested that Reb-A synthesis is governed by others factors not by growing conditions. Brandle [56] suggests that the presence of Reb-A is controlled by a single gene, but there may be an additive multiallelic locus for controlling the actual proportions. The functional role of the recombinant UGTSr in the synthesis of Reb-A was also ascertained by Madhav et al. [57].

The soil pH tended to decline with the increasing level of NPK fertilizer, which is in accordance with the finding of Dong et al. [58]. Thus, this result suggested that chemical fertilizer could increase soil acidity to some extent. Applied N PK fertilizer did not significantly alter the soil OC, AN, AP and AK. However, soil OC was increased to some extent with higher dose of N. These results may be due to the fact that higher level of N ensures the large and constant presence of active microorganism and the regular dynamic of biomass carbon [59]. In contrast, AN was declined with higher dose of applied N probably due to higher removal of N through aboveground biomass and high C/N ratio.

## Conclusions

The results, obtained in the present study, suggest that the dry leaf yield and biosynthesis of secondary metabolites of stevia are strongly controlled by the exogenous supply of plant nutrition, soil properties and climatic conditions of the growing region. Therefore, it can be concluded that higher dose of N and moderate level of K are helpful to increase the dry leaf yield under CSIR-IHBT and RHRS conditions. Furthermore, the sub-temperate climatic conditions of CSIR-IHBT are more favourable compared with other two locations in terms of leaf yield and secondary metabolites accumulation particularly when plant was grown during 13–15 MSW. These observations indicate that stevia plants are not able to cope with high temperatures coupled with low humidity during initial vegetative growth stages. It can also be concluded that dry leaf yield and stevioside accumulation are governed by environment and agronomic practices. However, Reb-A is controlled by others factors like genetic and enzymatic [56,57]. The changes in leaf yield and accumulation patterns of stevioside observed in response to different environmental conditions and nutritional variations provide leads for developing the strategies to increase the productivity of the stevia under different agro-climatic conditions. Thus, leaf yield and secondary metabolite profiles of the stevia can be improved through the selection of appropriate growing locations and proper nutrient management. However, further studies are required to standardize the planting date for different regions and to understand the relationship between plant nutrient and enzyme activities which are responsible for secondary metabolites synthesis.
